# Structural elucidation of some antimicrobial constituents from the leaf latex of *Aloe trigonantha* L.C. Leach

**DOI:** 10.1186/s12906-015-0803-4

**Published:** 2015-08-12

**Authors:** Mekdes Megeressa, Daniel Bisrat, Avijit Mazumder, Kaleab Asres

**Affiliations:** Department of Pharmaceutical Chemistry and Pharmacognosy, School of pharmacy, College of Health Sciences, Addis Ababa University, Addis Ababa, Ethiopia; Department of Pharmaceutical Technology, Noida Institute of Engineering and Technology, 19 Knowledge Park II, Institutional Area, Greater Noida, 201306 India

**Keywords:** *Aloe trigonantha*, Antimicrobial activity, 8-*O*-methy-7-hydroxyaloin A/B, Aloesin, Aloin A/B, Aloin-6’-*O*-acetate A/B, Disk diffusion

## Abstract

**Background:**

The incidents of drug resistant microorganisms and the need of treatments for newly emerging pathogens are of great concern to the global community. Our ability to treat infectious diseases is dependent on the development of new pharmaceuticals, and one potential source being medicinal plants with traditional claims. The leaves of *Aloe trigonantha* L.C. Leach, an endemic Ethiopian plant, are locally used for the treatment of infectious and inflammatory diseases. This study explores the potential of the latex of this plant and compounds isolated thereof for their *in vitro* antibacterial and antifungal properties.

**Methods:**

Analytical RP-HPLC and silica gel preparative TLC were used for identification and isolation of active constituents, respectively. Characterization of the compounds was based on UV, IR, HR-ESIMS, ^1^H and ^13^C NMR, and 2D-NMR spectral assignments. Antimicrobial activity studies were carried out against 21 pathogenic bacterial and 4 fungal strains using the disk diffusion method. Minimum inhibitory concentrations (MICs) were determined by the broth dilution method.

**Results:**

A *C*-glycosylated chromone identified as aloesin, and three *C*-glycosylated anthrones characterized as 8-*O*-methy-7-hydroxyaloin A/B, aloin A/B and aloin-6’-*O*-acetate A/B were isolated. The latex and isolated compounds exhibited *in vitro* antibacterial activity against the tested pathogens. In some cases the activity of the isolated compounds (MIC = 10 μg/mL) was comparable with that of the standard drug ciprofloxacin, particularly against some of the Gram-negative bacterial strains tested. However, their activity towards the fungal pathogens tested was relatively weaker showing maximum activity against *Candida albicans* with MIC value of 400 μg/mL.

**Conclusion:**

The present findings can be used for further research aimed at the development of new antibacterial agents, and may also justify the ethnomedicinal claim of the plant for the treatment of infectious diseases.

## Background

Microbial diseases are among the major causes of morbidity and mortality in the world, and particularly in developing countries [1, 2]. Regardless of the extensive works done towards the control of microorganisms, incidents of drug resistant microorganisms, reduction in new antimicrobials in pharmaceutical pipeline and the need of treatments for newly emerging pathogens pose an enormous threat to public health [3]. A report compiled by WHO indicates that devoid of urgent and coordinated action, the world is heading towards a post-antibiotic era in which common infections and minor injuries, which have been treatable for decades, can once again kill [4]. These negative health trends have driven a global initiative towards the search for new sources of antimicrobial substances mainly from medicinal plants. Medicinal plants represent rich sources of antimicrobials with higher activity and reduced side effects. It is, therefore, suggested to carry out an intense screening of these plants in order to validate their use in folk medicine and isolate the active principle(s) [5–7].

The genus *Aloe* is represented by 600 species and belongs to the family of Asphodelaceae [8, 9]. Aloe plants are native to sub-Saharan Africa, many islands of western Indian Ocean, including Madagascar and Saudi Arabian Peninsula. In Ethiopia and Eritrea, about 46 species of *Aloe* have been described so far with a high proportion of endemics adapted to harsh climates [10]. It has been reported that the leaf latex of several *Aloe* species and their constituents possess wide spectrum of biological activities, such as antimicrobial [11, 12], antimalarial [13], and antiglycation [14].

*Aloe trigonantha* L.C. Leach, locally called ‘Eret’, is among the endemic *Aloe* species of Ethiopia, which has been described in 1971 in an area between the cities of Bahir Dar and Gonder in the northern part of the country. Like many other *Aloe* species found in Ethiopia with ethnomedicinal applications [15, 16], the local people extensively use the leaves of *A. trigonantha* for the treatment of infectious and inflammatory diseases [17]. However, despite its use in traditional medicine and possible therapeutic applications, no phytochemical or biological studies carried out on the plant could be found in the literature. The purpose of this study is, therefore, to test the latex against a panel of microbes to characterize its antimicrobial effect. The report further details the extraction, isolation, identification and structural elucidation of the antimicrobial compounds of the latex.

## Methods

### Plant material

The latex of *A. trigonantha* was collected in January 2011 from Kutkuatema, a small village 568 km northwest of Addis Ababa, Ethiopia. The plant was authenticated by Professor Sebsebe Demissew, the National Herbarium, Department of Biology (DoB), Addis Ababa University (AAU) where voucher specimen was deposited with collection number MD 001.

### Bacterial strains

*In vitro* antibacterial assays were carried out against the following Gram-positive bacterial strains: *Bacillus pumilus* 82, *B. subtilis* ATCC 6633 and *Staphylococcus aureus* ML 267. The Gram-negative bacterial strains used were: *Escherichia coli* 3:37C*, E. coli* 7360, *E. coli* 872, *E. coli* CD/99/1, *E. coli* K 88, *E. coli* T 37, *E. coli* ROW 7/12, *E. coli* 5933, *Salmonella enterica* TD 01, *S. typhi* Ty2, *Shigella boydii* D13629, *S. dysentery* 8, *S. flexneri* Type 6, *S. soneii* 1, *Vibrio cholerae* NCTC 5596, *V. cholerae* NCTC 10732, *V. cholerae* NCTC 11501, and *V. cholerae* NCTC 4693. All the bacterial strains were procured from the Department of Technology, Jadavpur University; Central Drugs Laboratory, Kolkata and Institute of Microbial Technology, Chandigarh, India. The purity of the strains was checked according to the standard microbiological, cultural and biochemical tests prior to sensitivity tests against the test samples.

### Fungal strains

Antifungal activity testing was carried out on the following fungal pathogens: *Aspargillus niger* ATCC 6275, *Candida albicans* ATCC 10231, *Penicillium funiclosum* NCTC 287and *P. notatum* ATCC 11625. All the fungal strains were procured from Central Drugs Laboratory, Kolkata, India.

### Instrumental analysis

UV spectra were recorded on a Shimadzu Spectrophotometer MultiSpec-1501 (200-400 nm) at room temp. IR spectra were carried out in KBr plates on a Perkin-Elmer BX (400–4000 cm^−1^) instrument. HPLC was performed using an UltiMate 3000 Standard HPLC Systems equipped with a UV photodiode array detector (190–400 nm). ESI-MS were recorded on an Ultimate 3000 LC-MS with negative mode. The source voltage and temperature were fixed at 3 kV and 250 °C. NMR spectra were recorded on a Bruker Avance DMX400 FT-NMR spectrometer operating at 400 MHz for ^1^H and 100 MHz for ^13^C at room temperature. Signals were referred to an internal standard TMS. Chemical shifts were reported in δ units and coupling constants (*J*) in Hz.

### Extraction of the latex

The leaf latex of *A. trigonantha* was collected by cutting the leaves transversally near the base and arranging them concentrically around a plate. The latex was then left in open air for three days to allow evaporation of water, which yielded a dark brown powder.

### HPLC operating conditions

HPLC was performed using a Phenomenex IB-Sil C-18 reversed phase column (250 mm x 3.2 mm, 5 μm diameter particles); flow rate 1 mL/min. The solvent system consists of 30 % to 60 % linear gradient of methanol in water over 25 min, 3 min isocratic, 100 % in 2 min, 4 min isocratic. UV absorption spectra were recorded at 254 nm.

### Isolation of compounds

The latex was dissolved in methanol and applied directly to preparative thin layer chromatography (PTLC) plates over silica gel G6 F_254_ (Merck; 20 cm × 20 cm; 0.50 mm thickness). A solvent system of EtOAc/MeOH/H_2_O (77:13:10) was used for isolation. PTLC plates with 0.25 mm thickness were used to further purify the compounds. Chromatographic zones were visualized first in daylight and then under ultraviolet light of wavelengths 254 and 366 nm. The bands were scraped off, washed with EtOAc/MeOH (1:1) and filtered to yield four compounds (1–4).

- Aloesin (1): A pale yellow amorphous solid; 6.8 % (w/w); HPLC: *t*_*R*_ = 11.70 min; TLC: *R*_*f*_ = 0.32 (EtOAc/MeOH/H_2_O-77:13:10); UV λ_max_ (MeOH): 215, 244, 253, 295 nm; IR ν_cm-1_: 3436, 2923, 1684, 1384. LRESI-MS (−ve mode) *m/z*: 393[M-H]^−^, indicating a molecular formula of C_19_H_22_O_9_. ^1^H NMR (DMSO) δ: 2.25 (3H, *s*, 11-CH_3_), 2.62 (1H, *s*, 5-CH_3_), 3.15-3.70 (5H, *m*, H-2’-H-6’), 3.21 (1H, *s*, H-9), 4.70 (1H, *brs*, H’-1), 6.12 (1H, *s*, H-3), 6.69 (1H, *s*, H-6). ^13^C NMR (DMSO): 23.06 (5-CH_3_), 30.26 (11-CH_3_), 47.97 (9-CH_2_), 61.80 (C-6’), 73.88 (C-1’), 70.83 (C-4’), 71.10 (C-2’), 79.02 (C-2’), 81.56 (C-5’), 110.88 (C-8), 112.48 (C-3), 114.27 (C-4a), 117.97 (C-6), 147.84 (C-1a), 160.65 (C-7), 163.66 (C-2), 179.47 (C-4), 140.48 (C-5), 203.82 (C-10).
- 8-*O*-Methyl-7-hydroxyaloin A/B (2): A brown amorphous solid; 12.5 % (w/w); HPLC: *t*_*R*_ =16.87 min; TLC: *R*_*f*_ = 0.35 (EtOAc/MeOH/H_2_O-77:13:10); UVλ_max_ (MeOH): 199, 222, 294, 348 nm; IRν_cm-1_: 3411, 2853, 1636. HRESI-MS (+ve mode) *m/z*: 471.12640 [M + Na]^+^, (calcd. for C_22_H_24_O_10_ 471.12617 [M + Na]^+^)_._^1^H NMR (DMSO) δ: 1.2-3.5 (H-5’, *m*, H-2’- H-6’), 3.2 (1H, *brs*, H-1’), 3.81 (3H, *s*, 8-OCH_3_), 4.43 (1H, *d,* H-10), 4.48 (1H, *brs*, H-2), 6.75 (1H, *brs*, H-2), 6.78 (1H, *brs*, H-4), 7.05 (1H, *d*, H-6), 7.14 (1H, *d*, H-5), 8.41 (1H, *s*, 7-OH), 12.16 (1H, *s*, 1-OH). ^13^C NMR (DMSO) δ: 44.10 (C-10), 61.40 (8-OCH_3_), 62.10 (C-6’), 62.90 (3-OCH_2_), 70.90 (C-2’), 72.60 (C-4’), 78.40 (C-1’), 80.80 (C-3’), 84.20 (C-5’), 115.80 (C-4), 117.60 (C-5), 118.45 (C-8a), 120.80 (C-2), 125.30 (C-6), 127.90 (C-1a), 131.90 (C-4a), 140.86 (C-5a), 145.30 (C-7), 147.00 (C-8), 150.50 (C-3), 160.50 (C-1), 190.20 (C-9).
- Aloin A/B (3): A pale yellow amorphous powder; 15.7 % (w/w); HPLC: *t*_*R*_ = 22.19 min; TLC: *R*_*f*_ = 0.39 (EtOAc/MeOH/H_2_O-77:13:10); UV λ_max_ (MeOH) : 208, 299, 357 nm; IR ν_cm‑1_: 3447, 1631, 1618; LRESI-MS (−ve mode) *m/z*: 417 [M‑H]^−^, indicating a relative molecular weight (*M*_*r*_) of 418 (C_21_H_22_O_9_);^1^H and^13^C NMR chemical shift of compound 3 were in a good agreement with those reported for the same compound [11, 12].
- Aloin-6’-*O*-acetate A/B (4): A pale yellow amorphous solid; 18.4 % (w/w); HPLC: *t*_*R*_ = 23.87 min; TLC: *R*_*f*_ = 0.63 (EtOAc/MeOH/H_2_O-77:13:10); UV: λ_max_ (MeOH): 200, 269, 293 and 359 nm; IR ν_cm-1_: 3400, 2922, 1719, 1603, 1451 and 1383. HRESI-MS (+ve mode) m/z: 483.12624 [M + Na] ^+^, (calcd. for C_23_H_24_O_10_ 483.12617 [M+ Na] ^+^). ^1^H NMR (DMSO) δ: 1.2-3.7 (5H, *m*, H-2’- H-6’), 2.1 (3H, *s*, COCH_3_), 3.21 (1H, *dd*, H-1’), 4.59 (2H, *d*, 11-CH_2_), 4.83 (1H, *brs*, H-10), 6.76 (1H, *s*, H-2), 6.97 (1H, *brs*, H-7), 7.23 (1H, *brs*, H-4), 7.38 (1H, *d*, H-5), 7.43 (1H, *dd*, H-6), 11.79 (1H, *s*, 1-OH), 11.80 (1H, *s*, 8-OH).^13^C NMR (DMSO) δ: 21.20 (C-7’), 44.50 (C-10), 61.80 (C-11), 65.03 (C-6’), 70.68 (C-2’), 70.70 (C-4’), 78.60 (C-3’), 81.30 (C-5’), 85.4 (C-1’), 114.20 (C-2), 115.00 (C-1a), 115.90 (C-7), 117.80 (C-8a), 119.20 (C-4), 120.70 (C-5), 136.80 (C-6), 142.30 (C-4a), 144.7 (C-5a), 150.50 (C-3), 161.20 (C-1), 161.30 (C-8), 170.80 (C-8’), 193.75 (C-9).

### *In vitro* antibacterial activity test

*In vitro* antibacterial assay was screened by the disc diffusion method as described by Mitchell and Carter [18], by determining zones of inhibition produced by the test samples and comparing them with those of ciprofloxacin. A stock solution of the latex and various isolated compounds was prepared in 1 % DMSO (1 mg/ml) for the antibacterial activity tests and 10 mg/ml for the antifungal activity tests. Concentrations ranging from 5 to 800 μg/mL for antibacterial activity tests, and 50 to 2000 μg/mL for antifungal activity tests were employed by diluting the stock solution in appropriate volumes of water. Serial nutrient agar plates were then prepared by pouring of molten media into sterile Petri dishes and incubated at 37 °C for 24 h to check for any sort of contamination. The inoculums were then swabbed uniformly and allowed to dry for 5 min. Then, the filter paper discs (Whatman no. 1) of 6 mm were impregnated with the test samples and placed on the surface of the medium. The Petri dishes were then incubated at 37 °C for 24 h and the diameter of zone of inhibition was measured in mm. Similar procedure was adopted for ciprofloxacin and the corresponding zone of inhibition was compared accordingly. 1 % DMSO was used as a negative control.

### *In vitro* antifungal activity test

The antifungal potential of the test samples (2000 μg/mL) was screened by disc diffusion method (as described for the determination of antibacterial activity) against the fungal pathogens on Saborauds dextrose media. The Petri dishes were incubated at room temperature for 3 days and the diameter of zone of inhibition was measured in mm. Griseofulvin was used as a reference standard.

### Determination of minimum inhibitory concentrations (MICs)

The MICs of the latex and isolated compounds were determined by the broth dilution method, as described by Hecht *et al*. [19]. Nutrient agar and Saborauds dextrose agar were used for bacterial and fungal growth, respectively. Concentrations ranging from 5 to 800 μg/mL for antibacterial activity tests, and 50 to 2000 μg/mL for antifungal activity tests were employed by dissolving the test samples in DMSO. A sterility control was also carried out (growth control contained nutrient broth plus DMSO, without antimicrobial substances). Each test and growth control well was incubated for 24 h at 37 °C for bacteria and 3 days at 25 °C for fungi.

## Results and discussion

### Structure elucidation

**Fig. 1:**
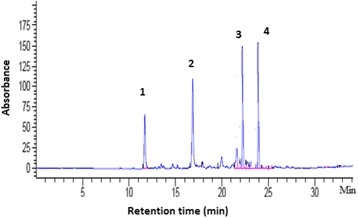
HPLC chromatogram of the leaf latex of *Aloe trigonantha*. Column: RP-18 (250 mm x 3.2 mm, 5 μm diameter particles); flow rate 1 mL/min; UV detection: 254 nm; Solvent system: 30 % to 60 % linear gradient of methanol in water over 25 min, 3 min isocratic, 100 % in 2 min, 4 min isocratic

Reversed phase-HPLC analysis of the leaf latex of *A. trigonantha* revealed the presence of four major compounds (Fig. 1), identified as aloesin (1), 8-*O*-methy-7-hydroxyaloin A/B (2) aloin A/B (3) and aloin-6’-*O*-acetate A/B (4) by spectroscopic techniques including ESIMS, ^1^H, ^13^C NMR and DEPT-135 spectral data.

**Fig. 2:**
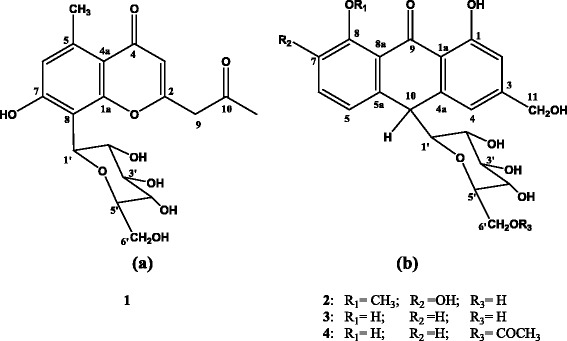
Structures of (**a**) aloesin (1); (**b**) 8-*O*-methyl-7-hydroxyaloin A/B (2), aloin A/B (3) and aloin-6’-*O*-acetate A/B (4)

Compound 1, isolated as a pale yellow amorphous solid, showed [M-H]^−^ at *m/z* = 393 in the negative-mode LRESI-mass spectrum, indicating a relative molecular weight of 394. A molecular formula of C_19_H_22_O_9_ was deduced based on LRESI-MS, ^1^H and ^13^C and DEPT 135 spectral data. The presence of a chromone skeleton was deduced from the UV spectrum (λ_max_: 215, 244, 253 and 295 nm) [20], ^1^H and ^13^C NMR spectral data. Hence, compound 1 was unequivocally identified as 2-acetonyl-8-*β*-D-glucopyranosyl-7-hydroxy-5-methylchromone, commonly known as aloesin (Fig. 2) by comparing its ^1^H and ^13^C NMR data with those reported for the same compound in the literature [21].

Compound 2 was isolated as a brown amorphous solid. The positive-mode HRESI-mass spectrum of compound 2 gave a pseudomolecular ion at *m/z* 471.12640 [M + Na]^+^, corresponding to a molecular formula of C_22_H_24_O_10_ (calcd. 471.12617)_._ It was identified as an anthrone derivative from the UV spectrum (λ_max_: 199, 222, 294 and 348 nm), ^1^H and ^13^C NMR spectral data [20]. The^1^H and ^13^C NMR spectral data of compound 2 revealed that the signals mostly appear either in pairs or overlapping on one another further suggesting that the compound is a mixture of two closely related compounds. Hence, compound 2 was unambiguously characterized as the known 8-*O*-methyl-7-hydroxyaloin A/B (10-C-*β*-D-glucopyranosyl-1,7-dihydroxy-8-*O*-methyl-9-anthracen-one) (Fig. 2) by comparing its ^1^H and ^13^C NMR spectra data (listed in the Method section) with those reported for the related compound, homonataloin [22].

Compound 3 was obtained as a yellow amorphous solid with a pseudomolecular ion at *m/z*: 417 [M-H]^−^ in negative-mode ESI-mass spectrum. A molecular formula of C_21_H_22_O_9_ was determined based on its ESI-MS, ^1^H and ^13^C NMR spectral data. Thus, compound 3 was unequivocally characterized as aloin A/B, which was previously isolated from a number of *Aloe* species, by comparing its ^1^H and ^13^C NMR spectral data with those data reported in the literature for the same compound [11, 12].

Compound 4 was obtained as a yellow amorphous solid with the positive-mode HR-ESI mass spectrum showing a pseudomolocular ion at *m/z* = 483.12624 [M + Na] ^+^, indicating a molecular formula of C_23_H_24_O_10_ (calcd. 483.12617). The presence of an anthrone moiety in compound 4 was confirmed from its UV (λ_max_: 200, 269, 293 and 359 nm), ^1^H and ^13^C NMR spectral data. A close analysis of the ^1^H and ^13^C NMR spectra revealed that the signals occurred in pairs, indicating that it was a mixture of two closely related compounds. Consequently, from its spectral data summarized in the Method section, and by comparing this data with those previously reported for the same compound [23], compound 4 was identified as 10-C-*β*-D-glucopyranosyl-1,8-dihydroxy-3-hydroxymethyl-6’-*O*-acetate-9(10H)-anthracenone, otherwise known as aloin-6’-*O*-acetate A/B (Fig. 2). Literature survey indicates that aloin-6’-*O*-acetate A/B (4) is a rare natural product being previously isolated only once from the leaf latex of *Aloe trichosantha* [23].

### Antibacterial activity

In the present study, the leaf latex of *A. trigonantha* was evaluated against 21 bacteria strains using the disc diffusion method. Our experiments showed that the latex possesses activity against most of the bacterial strains tested (Table 1). Whilst the Gram-negative bacteria including all the strains of *E. coli* and *Salmonella* spp were particularly susceptible to the latex*,* the Gram-positive rod bacteria *Bacillus pumilus* and *B. subtilis* appeared to be resistant. *In vitro* antibacterial activity of the leaf latex of *Aloe harlana* had previously been examined in our laboratory showing the strongest activity against several Gram-negative bacterial strains [11]. Similarly, Mariappan and Shanthi [24] reported the inhibition of *Salmonella* spp, *Shigella sonie* and *Staphylococcus* spp by the leaf latex of *Aloe vera* with MIC values of 12.3, 4.2 and 12.4 μg/mL, respectively.

**Table 1:** Zones of inhibition and minimum inhibitory concentrations (MICs) of the latex and compounds isolated from *Aloe trigonantha*

Microorganism		Zones of inhibition in mm (200 μg/mL)						MIC (μg/mL)^i^			
	Latex^ii^	1^ii^	2^ii^	3 ^ii^	4^ii^	Cipro	Latex	1	2	3	4
*Bacillus pumilus* 82	6.0 ± 0.0	6.0 ± 0.0^a^	6.0 ± 0.0^a^	10.0 ± 0.5^b^	6.0 ± 0.0^a^	19.0 ± 1.3	-	-	-	200	-
*B. subtilis* ATCC 6633	6.0 ± 0.0	6.0 ± 0.0^a^	6.0 ± 0.0^a^	10.0 ± 0.6^b^	6.0 ± 0.0^a^	18.0 ± 0.7	-	-	-	200	-
*Escherichia coli* 3:37C	15.0 ± 0.9	15.0 ± 1.2^a^	15.0 ± 0.9^a^	14.5 ± 0.5^a^	15.0 ± 0.7^a^	16.5 ± 0.8	50	25	25	10	25
*E. coli* 872	15.0 ± 0.7	14.5 ± 0.9^a^	14.5 ± 0.6^a^	14.5 ± 0.4^a^	14.5 ± 1.2^a^	16.0 ± 0.9	50	25	25	10	25
*E. coli* CD/99/1	15.0 ± 0.8	15.0 ± 0.7^a^	15.0 ± 0.8^a^	15.5 ± 0.9^a^	15.0 ± 0.9^a^	17.0 ± 1.4	50	25	25	10	25
*E. coli* K88	16.5 ± 1.1	15.5 ± 0.5^b^	15.5 ± 1.2^b^	15.0 ± 1.0^b^	15.5 ± 0.9^b^	17.0 ± 0.5	50	50	25	10	25
*E. coli* LT37	15.5 ± 0.6	14.5 ± 0.4^b^	14.5 ± 1.1^b^	14.5 ± 1.0^b^	14.5 ± 0.5^b^	16.0 ± 0.8	50	25	25	10	25
*E. coli* NCTC 5933	15.5 ± 1.2	15.0 ± 0.9^a^	14.5 ± 0.9^b^	14.5 ± 0.5^b^	15.0 ± 0.8^a^	16.0 ± 0.6	50	50	50	10	25
*E. coli* NCTC 7360	16.5 ± 1.0	15.5 ± 1.1^b^	14.5 ± 0.6^b^	14.5 ± 0.7^b^	15.5 ± 0.7^b^	17.0 ± 1.3	50	50	50	10	25
*E. coli* ROW 7/12	15.5 ± 0.7	15.0 ± 0.9^a^	15.0 ± 0.5^a^	14.5 ± 0.2^a^	15.0 ± 0.6^a^	16.5 ± 0.4	50	25	25	10	25
*Salmonella enterica* TD 01	16.5 ± 0.9	15.0 ± 0.6^b^	13.5 ± 0.8^b^	12.5 ± 0.5^b^	16.0 ± 1.2^a^	19.0 ± 0.9	50	50	100	50	25
*S. typhi* Ty2	15.0 ± 1.2	15.0 ± 0.8^a^	14.0 ± 0.7^b^	14.0 ± 0.6^b^	15.0 ± 0.9^a^	16.0 ± 0.5	50	25	50	10	25
*Shigella boydii* D 13629	15.0 ± 0.5	15.5 ± 0.8^a^	14.5 ± 0.9^a^	16.5 ± 0.4^a^	15.5 ± 0.6^a^	20.0 ± 0.7	50	50	50	10	50
*S. dysentery* 8	14.5 ± 0.8	14.0 ± 0.7^a^	13.5 ± 0.9^b^	15.5 ± 0.6^b^	13.5 ± 0.4^b^	20.0 ± 0.8	50	25	50	10	50
*S. flexneri* Type 6	15.0 ± 0.7	15.5 ± 0.5^a^	15.5 ± 0.6^a^	15.5 ± 0.5^a^	15.5 ± 0.7^a^	20.5 ± 0.7	50	50	50	10	50
*S. soneii* 1	15.0 ± 0.5	15.5 ± 0.9^a^	15.5 ± 0.8^a^	15.5 ± 0.5^a^	15.5 ± 0.6^a^	19.5 ± 1.2	50	100	50	10	50
*Staphylococcus aureus* ML 267	14.5 ± 0.9	14.5 ± 1.3^a^	14.5 ± 0.5^a^	14.5 ± 0.6^a^	13.5 ± 0.9^b^	18.0 ± 1.4	100	50	100	25	100
*Vibrio cholerae* NCTC 10732	14.0 ± 0.4	15.5 ± 0.8^b^	15.0 ± 1.1^b^	14.5 ± 1.0^b^	15.5 ± 0.9^b^	19.0 ± 0.9	50	25	25	10	25
*V. cholerae* NCTC 11501	14.5 ± 0.6	15.5 ± 0.4^b^	14.0 ± 0.7^a^	15.0 ± 0.3^a^	15.5 ± 0.7^b^	18.5 ± 0.8	50	25	25	10	25
*V. cholerae* NCTC 4693	13.5 ± 0.8	14.5 ± 0.9^b^	15.0 ± 0.8^b^	15.0 ± 0.3^b^	14.5 ± 0.9^b^	17.5 ± 0.5	50	25	25	10	25
*V. cholerae* NCTC 5596	14.0 ± 0.3	15.0 ± 0.7^b^	15.0 ± 0.7^b^	15.0 ± 0.5^b^	15.0 ± 0.5^b^	18.5 ± 0.8	50	25	25	10	25
Fungal strains	Latex^ii^	1^ii^	2^ii^	3^ii^	4^ii^	Gris.	Latex	1	2	3	4
*Aspergillus niger* ATCC 6275	9.0 ± 0.7	11.0 ± 1.3^b^	12.0 ± 0.9^b^	12.5 ± 0.6^b^	12.5 ± 0.9^b^	15.0 ± 0.9	1500	1000	1000	800	1000
*Candida albicans* ATCC 10231	10.0 ± 0.9	10.0 ± 0.8^a^	14.0 ± 1.2^b^	13.5 ± 1.0^b^	14.5 ± 0.8^b^	16.0 ± 1.3	800	400	400	800	400
*Penicillium funiculosum* NCTC 287	8.0 ± 0.6	9.5 ± 0.5^a^	12.5 ± 0.7^b^	12.0 ± 0.4^b^	13.5 ± 0.5^b^	13.5 ± 1.1	1000	800	800	800	400
*P. notatum* ATCC 11625	8.0 ± 0.8	10.0 ± 0.7^b^	12.5 ± 0.6^b^	12.0 ± 0.5^b^	12.5 ± 0.4^b^	13.5 ± 0.9	1000	800	800	800	400

^i^MIC expressed as an average from three independent experiments, each performed in triplicate; ^ii^Zones of inhibitions were expressed as means ± SEM including the 6 mm diameter of the disc; ^a^: *p* > 0.05 when compared to the latex on the same row; ^b^: *p* < 0.05 when compared to the latex on the same row; 1: Aloesin, 2: 8-*O*-Methyl-7-hydroxyaloin A/B, 3: Aloin A/B; 4: Aloin-6’-*O*-acetate A/B; Cipro: Ciprofloxacin; Gris: Griseofulvin

In view of the promising antimicrobial growth inhibitory effect of the latex, it was further subjected to compound isolation with the aim of finding more potent antibacterial agent(s). As shown in Table 1, the isolated compounds inhibited growth of most of the bacterial strains tested showing lower MIC values against most strains of *E. coli* and *V. cholera* than that of the latex. In particular, aloin A/B (3) showed potent action against all strains with the exception of *Bacillus* spp., and *Salmonella enterica*, which showed limited susceptibility. Aloin isolated from the latex of *A. trigonantha* also showed powerful inhibitory activity and its action was similar to aloin isolated from the latex of *A. sinana* reported in our previous work [12]. In general, the antibacterial profile of the latex was similar to those of the isolated compounds, being able to inhibit mainly the growth of Gram-negative bacteria. It appears that there was synergy among the isolated compounds as the MIC values of the latex was smaller than those of compounds 1 and 2 against *Shigella soneii* and *Salmonella enterica*, respectively. In spite of the fact that components of the latex belong to two different classes of secondary metabolites, their antibacterial activity was comparable with each other. Aloesin (1) is a chromone while the others are anthrone glycosides. Previous studies have demonstrated that natural chromones isolated from diverse plant species such as *Cassia petersiana* (Fabaceae) [25], *Ferula communis* (Apiaceae) [26] and *Aloe barbadensis* (Asphodelaceae) [27] as well as synthetic phosphonic derivatives of chromones [28] have activity towards Gram-negative and Gram-positive bacteria. Similarly, anthraquinones which are structurally related to anthrones have been shown to inhibit growth of several pathogenic microorganisms [29, 30].

The mechanism of action by which chromones and anthrones exert their antibacterial action is not well studied. Whilst Hamman [31] proposed that the antibacterial activity of anthraquinones isolated from the latex of *A. vera* is through mediation of solute transport inhibition in membranes, Ubbink-Kok *et al*. [32] reported that emodin and emodin anthrone are active against *E. coli* by inhibiting respiration-driven transport within membranes. Similar mechanisms of action could be proposed for 8-*O*-methyl-7-hydroxyaloin A/B (2), aloin A/B (3) and aloin-6’-*O*-acetate A/B (4) owing to their structural similarity to anthraquinones. However, further studies are needed to establish the actual mechanism(s) of action for these classes of compounds.

Among the bacterial strains tested, only the Gram-positive bacilli bacteria were found to be resistant to the latex and its components. Similarly, the single Gram-positive strain of *S. aureus* tested in the present study was shown to have a limited susceptibility to both the latex and its components. It is interesting to note that nearly all the Gram-negative pathogens tested were susceptible to both the latex and isolated compounds although they have phospholipid membrane carrying the structural lipopolysaccharide component that makes their cell wall impermeable to antimicrobial substances. This situation can be explained in terms of the chemical nature of the test samples, which could have an effect on the permeability and integrity of the bacterial cell wall. According to Alves *et al*. [33], the anthraquinones emodin and barbaloin affect the phospholipid membranes of bacterial cell wall resulting in remarkable changes in membrane physical properties including changes of the lipid/water interface in negatively charged phospholipids and perturbations of the core of the phospholipid bilayer.

### Antifungal activity

The fungi tested in the present study were shown to have limited susceptibility to both the latex and its components although zones of inhibition displayed by the latex were somewhat smaller than those obtained for the isolated compounds. Among the fungal strains tested, *C. albicans* was found to be more susceptible (MIC = 400–800 μg/mL) to the test compounds than the other fungal pathogens, which exhibited different levels of sensitivity. Previous studies in our laboratory have found the anthrones aloin and 7-*O*-methylaloeresin isolated from the leaf latex of *A. harlana* to be capable of inhibiting the growth of *C. albicans*, *Penicillium funiculosum* and *P. notatum* to a similar extent observed in the present study [11]. Moreover, aloin from *A. ferox*, emodin and barbaloin from *Rheum australe* were shown to inhibit the growth of *C. albicans* [34] and a range of pathogenic fungal strains [35], respectively.

## Conclusions

The findings of the present study have established the susceptibilities of a broad range of bacteria particularly Gram-negative pathogens to the latex of *A. trichosantha* and its components. The Gram-positive strains tested were either not inhibited by or showed limited susceptibility to any of the tested substances. The inhibitory effects of the isolated compounds against various pathogenic microorganisms had clearly demonstrated the usefulness of *A. trichosantha* in the treatment of various diseases caused by these pathogenic strains and explains, in part or in whole, the use of the plant in traditional medicine. Furthermore, the identification of such natural antimicrobial compounds may lead to the development of novel antimicrobials through structure/activity studies.
